# PReS13-SPK-1582: Activity and damage - we have to measure them

**DOI:** 10.1186/1546-0096-11-S2-I23

**Published:** 2013-12-05

**Authors:** BM Feldman

**Affiliations:** 1 Pediatrics, Hospital For Sick Children, Toronto, Canada

## 

"You can't manage what you don't measure."

The world community--led by the IMACS and PRINTO groups--has developed robust core criteria to measure disease activity and damage for Juvenile Dermatomyositis studies. These measures are tremendously useful in clinical practice, and not just for research.

More problematic has been determining clinically meaningful change. The current definitions of change may not be adequate for clinical trials. Problems include mathematical theoretical ones (e.g. using percent [relative] change for non-ratio measures, and trying to calculate continuous scores from ordinal measures), and practical ones (not incorporating patients' subjective assessments properly).

Proper measures have an important place in the clinical care of children with dermatomyositis; we have made a very strong start, but there is still work to do.

"You can't manage what you don't measure."

The world community--led by the IMACS and PRINTO groups--has developed robust core criteria to measure disease activity and damage for Juvenile Dermatomyositis studies. These measures are tremendously useful in clinical practice, and not just for research.

More problematic has been determining clinically meaningful change. The current definitions of change may not be adequate for clinical trials. Problems include mathematical theoretical ones (e.g. using percent [relative] change for non-ratio measures, and trying to calculate continuous scores from ordinal measures), and practical ones (not incorporating patients' subjective assessments properly).

Proper measures have an important place in the clinical care of children with dermatomyositis; we have made a very strong start, but there is still work to do.

## Disclosure of interest

None declared.

